# Urinary Fluoride Levels among Canadians with and without Community Water Fluoridation

**DOI:** 10.3390/ijerph18126203

**Published:** 2021-06-08

**Authors:** Julia K. Riddell, Ashley J. Malin, Hugh McCague, David B. Flora, Christine Till

**Affiliations:** 1 Department of Clinical Health Psychology, University of Manitoba, Winnipeg, MB N3E 3N4, Canada; 2 Department of Preventive Medicine, Keck School of Medicine of USC, Los Angeles, CA 90032, USA; ashley.malin@usc.edu; 3 Institute for Social Research, York University, Toronto, ON M3J 1P3, Canada; hmccague@yorku.ca (H.M.); dflora@yorku.ca (D.B.F.); 4 Faculty of Health, York University, Toronto, ON M3J 1P3, Canada; ctill@yorku.ca

**Keywords:** urinary fluoride, fluoride excretion, community water fluoridation, Canadian Health Measures Survey (CHMS)

## Abstract

Drinking water is a major source of dietary fluoride intake in communities with water fluoridation. We examined the association between urinary fluoride adjusted for specific gravity (UF_SG_) and tap water fluoride levels, by age and sex, among individuals living in Canada. Participants included 1629 individuals aged 3 to 79 years from Cycle 3 (2012–2013) of the Canadian Health Measures Survey. We used multiple linear regression to estimate unique associations of tap water fluoride levels, age, sex, ethnicity, body mass index (BMI), use of fluoride-containing dental products, smoking in the home, and tea consumption with UF_SG_. UF_SG_ concentration was significantly higher among participants who received fluoridated drinking water (*mean* = 1.06 mg/L, *standard deviation* = 0.83) than among those who did not (*M* = 0.58 mg/L, *SD* = 0.47), *p* < 0.01. UF_SG_ increased over adulthood (ages 19 to 79). Higher UF_SG_ concentration was associated with being female, tea drinking, and smoking in the home. In conclusion, community water fluoridation is a major source of contemporary fluoride exposure for Canadians. Lifestyle factors including tea consumption, as well as demographic variables such as age and sex, also predict urinary fluoride level, and are therefore important factors when interpreting population-based fluoride biomonitoring data.

## 1. Introduction

Fluoride has been added to public drinking water supplies since the 1940s for prevention of dental caries. Approximately 73% of the U.S. population using public drinking water systems receives fluoridated water compared with 39% of Canadians [1] and only 3% of Europeans [2,3]. In the U.S. and Canada, 0.7 mg/L is the recommended concentration of fluoride in drinking water for the prevention of dental caries [4,5]. While fluoridated drinking water is considered the main source of dietary fluoride intake [6,7], other sources can include dental products, supplements, and dietary products that contain naturally occurring fluoride, such as tea [8,9,10], or foods that are sprayed with fluoride-containing pesticides (e.g., grapes).

Most population-based biomonitoring studies examining fluoride exposure (e.g., [11,12,13]) provide nationally or provincially representative reference values for urinary fluoride levels that are not separated by community water fluoridation (CWF) status. However, water fluoride concentrations are moderately to strongly correlated with fluoride levels in urine [14,15,16,17] and blood plasma [18,19,20]. Levels of urinary fluoride are approximately 1.5 to 2 times higher in fluoridated regions than in non-fluoridated regions [14,17,21,22]. Because water fluoridation is known to be a major source of fluoride, it is important to analyze fluoride excretion levels by CWF status, particularly in populations vulnerable to potential adverse health effects of fluoride exposure.

Fluoride excretion patterns may also differ by age and sex due to differences in fluoride intake and distribution in mineralized tissues [5,18,23,24,25,26]. We characterized urinary fluoride levels according to age, sex, and CWF status in a large sample of individuals aged 3 to 79 years living in Canada. We also examined predictors of urinary fluoride levels, including ethnicity, body mass index (BMI), use of fluoride-containing dental products, smoking in the home, and consumption of tea and fluoridated tap water, controlling for income and highest household education.

## 2. Methods

### 2.1. Data Source and Participants

We used data from Cycle 3 (2012–2013) of the Canadian Health Measures Survey (CHMS) collected by Statistics Canada. All aspects of the CHMS were reviewed and approved by Health Canada’s Research Ethics Board [27]; the current study was approved by the York University Research Ethics Board (certificate: 2016-236).

The CHMS randomly selected participants aged 3 to 79 years who live in private households across Canada. Analyses were based on 2671 participants for whom tap water fluoride and urinary fluoride data were available (the urine and tap water subsample represented 46% of the full CHMS sample). Household tap water samples were collected during the initial visit to the home when the household questionnaire was completed. Urine samples were collected at a mobile lab [28]. Full details can be found at www.statcan.gc.ca (accessed on 7 June 2021).

**Figure 1:**
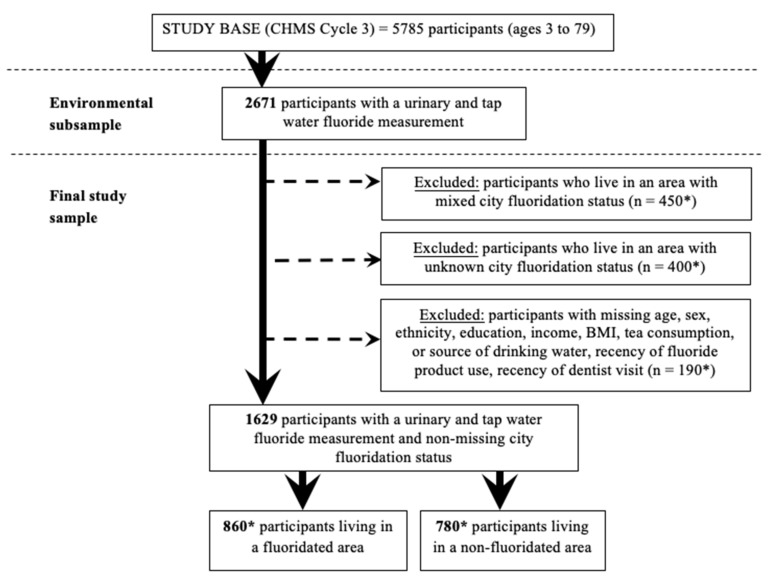
Flow chart of eligible participants in the study. Note: CHMS, Canadian Health Measures Survey; BMI, body mass index. * Signifies that the number has been rounded due to Statistics Canada vetting requirements.

Community water fluoridation (CWF) status was determined by viewing reports on each city’s website or contacting the water treatment plant (see Supplemental Table S1). Of the 16 sites, five received CWF and five did not receive CWF at the time of CMHS Cycle 3, corresponding to approximately 860 (33%) and 780 (30%) of 2617 participants with water fluoride data, respectively (rounded due to Statistics Canada data release requirements). Three sites were considered to have mixed fluoridation status due to the following reasons: unclear site boundaries (Southwest Montérégie, Quebec ~150 participants), some water treatment plants within the site added fluoride while others did not (West Montreal, Quebec ~150 participants), or water fluoridation stopped during the CHMS data collection period (Windsor, Ontario ~150 participants). An additional three sites were considered to have questionable fluoridation status due to having a mean water fluoride that was significantly different than other sites in the same category (fluoridated or non-fluoridated) likely to be due to naturally occurring fluoride and/or a large proportion of individuals in the sample using well water. Calgary, Alberta, which was said to be non-fluoridated at the time of data collection, was classified as having questionable fluoridation status because the average tap water level was three times higher than the average of all other non-fluoridated sites. Brantford-Brant County, Ontario and Kent Country, New Brunswick were said to be fluoridated based on online information (see Supplemental Table for websites), but for the purpose of this study they were classified as having questionable fluoridation status because the average tap water level for each site was three times lower than the average of all other fluoridated sites. See Figure 1 for a flow chart of how participants were included in the final analysis sample.

### 2.2. Measurement of Water Fluoride Concentration

Tap water samples were collected at respondents’ homes. Samples were analyzed for fluoride concentrations (mg/L) via a basic anion exchange chromatography procedure with a limit of detection (LoD) of 0.006 mg/L [29]. Concentrations at the LoD were assigned a missing value code by Statistics Canada, and these values were subsequently replaced with an imputed value of LoD/√2 (as recommended by [30]); 435 of 1629 (27%) water samples had fluoride levels below the LoD.

### 2.3. Measurement of Urinary Fluoride Concentration

Urine spot samples were collected under normal (non-fasting) conditions and were not standardized with respect to collection time. When tested, the correlation between time of day and UF_SG_ was near zero (r = −0.03). Fluoride concentrations in spot urine samples were analyzed using an Orion pH meter with a fluoride ion selective electrode after being diluted with an ionic adjustment buffer [31]. Urinary analyses were performed at the Human Toxicology Laboratory of the Institut National de Santé Publique du Québec (INSPQ; accredited under ISO 17025) under standardized operating procedures [31]. The precision and accuracy of the fluoride analyses, including quality control measures and quality assurance reviews, are described in previous publications [12]. The LoD for urinary fluoride was 10 μg/L for Cycle 3 [12]. No urinary fluoride values were below the LoD. Urinary fluoride concentrations were adjusted for specific gravity (UF_SG_; mg/L); specific gravity shows no systematic variation within a given day and is less dependent on body size, age, and sex than creatinine [32,33,34,35].

### 2.4. Drinking Water Habits

Participants were asked the following questions: When you drink water at home, what is your primary source of drinking water? (response options included tap water, bottled water, or other); and What is the source of the tap water in this home? (response options were municipal, private well, or other). Of the total sample of 1629, 461 participants (28%) did not answer these questions on drinking water habits. Of the remaining 1168 participants, approximately 930 (80%) reported drinking primarily tap water at home and 197 (17%) reported drinking primarily bottled water at home.

### 2.5. Other Sources of Fluoride Exposure

Regarding tea consumption, 670 of 1629 participants (41%) stated that they drink green, black, or white tea at least once per year. These individuals were asked follow-up questions including the number of cups they typically drink at a time when they do drink tea (response options were less than one cup, one to two cups, or more than two cups) and the last time they drank green, black, or white tea (within 24 h of the urinary fluoride sample collection or more than 24 h ago).

Participants were asked about the last time they used a fluoride-containing dental product. Due to the short half-life of fluoride, the response options were combined to create a binary variable (less than 6 h ago = 1, 6 or more hours ago = 2). Participants were also asked about the last time they received fluoride treatments at the dentist; the response options were again combined to create a binary variable (less than 3 months ago = 1, 3 months ago or more = 2).

### 2.6. Statistical Analysis

A small number of cases were identified as influential outliers based on a Cook’s Distance greater than 4/N. The outliers had the largest values of UF_SG,_ which were approximately seven times greater than the mean of their age group (the exact Cook’s Distance values and UF_SG_ values cannot be reported due to Statistics Canada policies regarding the release of individual data points). We removed these outlying cases from all analyses because they are unlikely to reflect plausible chronic exposure values. The highest incomes were identified as extreme observations based on high residuals; these values were replaced with the next highest income value (only 0.01% of income values were adjusted). Consistent with previous publications (e.g., [12,18]), we describe UF_SG_ values for six age groups: 3 to 6, 7 to 11, 12 to 18, 19 to 39, 40 to 59, and 60 to 79 years. We used Spearman rank correlations to describe the relationship between UF_SG_ and tap water fluoride levels using these age groups. For all other analyses, age was treated as a continuous variable. We used independent samples Welch’s *t*-tests to test whether UF_SG_ differed by sex or CWF status.

We used linear regression to predict UF_SG_ levels across the lifespan. Regression models included both dietary and dental sources of fluoride exposure (CWF status, tap water fluoride concentration, tea consumption, primary source of drinking water, time since last fluoride treatment at dentist, and time since use of a fluoride-containing dental product) and demographic variables that may affect fluoride metabolism or excretion including age, sex, ethnicity (white or ethnic minority), body mass index (BMI), and exposure to tobacco smoke [36]. Given that 650 participants (40%) reported that they do not drink tea, tea consumption was coded as a binary variable (yes = 1, no = 0) in the regression analysis. Finally, we included highest household level of education (less than a bachelor’s degree vs. bachelor’s degree or greater) and total household income (per $1000 Canadian) to control for any factors related to socioeconomic status. Plots of residuals by fitted values and plots of residuals against predictors and covariates were examined. A quadratic age effect was included in the regression model to test for a non-linear relation between age and UF_SG_; the residual plots showed no other concerns with non-linearity, non-normality, or non-constant variance. We also tested three two-way interactions: age by sex, age^2^ by sex, and CWF by primary source of drinking water (tap or bottled). Variance inflation factor (VIF) statistics indicated no concerns regarding multicollinearity [37]. A two-sided α = 0.05 was used as the threshold for statistical significance. The current study did not apply survey weights provided by Statistics Canada because this project was an extension of a previously published study examining individual level exposures and outcomes [22] that did not apply weights.

## 3. Results

### 3.1. Population Characteristics

The study sample had an approximately equal proportion of males (49%) and females (51%). The mean age was 32 years old, 73% were white, and 50% of the sample reported a high school, trade school, or college degree, while the other 50% reported a university degree or higher. The mean household income was $87,700 (median = $73,000) and the mean BMI was 24. Most demographic variables, including sex, age, and highest household education, had less than 5% missing data; 11% of participants had missing ethnicity and 15% of participants did not report either height or weight needed to calculate BMI.

Approximately half of the participants (53%) in the analytic sample with a UF_SG_ and tap water measurement lived in a region that adds fluoride to municipal tap water. The mean UF_SG_ concentration for the entire sample was 0.83 mg/L (SD = 0.72, median = 0.63) and the mean water fluoride concentration was 0.29 mg/L (SD = 0.29, median = 0.12). UF_SG_ and tap water fluoride concentrations were moderately correlated overall (*r* = 0.31, *p* < 0.05). Correlations between UF_SG_ and tap water fluoride concentrations were of the largest magnitude for ages 12 to 18 (*r* = 0.35), 19 to 39 (*r* = 0.42), 40 to 59 (*r* = 0.44), and 60 to 79 (*r* = 0.36) compared with children aged 3 to 6 (*r* = 0.18) and 7 to 11 (*r* = 0.24) years; all *p*-values < 0.05.

### 3.2. Urinary Fluoride Levels by Demographic Characteristics

**Figure 2:**
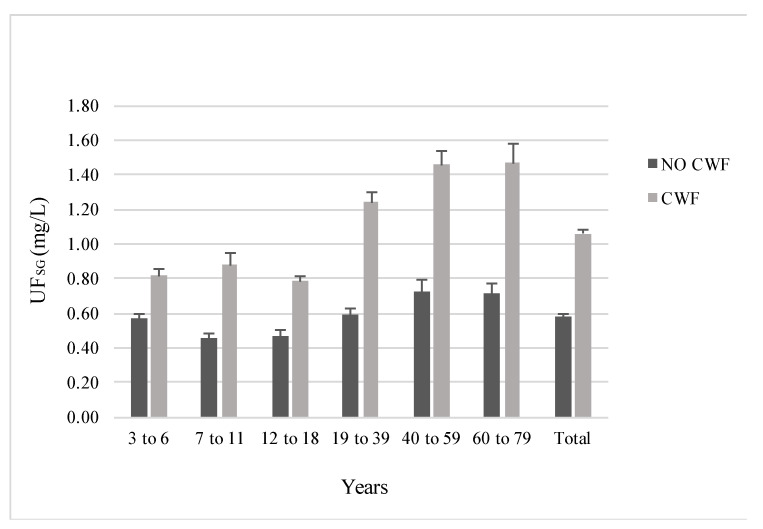
Mean levels of UF_SG_ by age group and CWF status. Error bars represent the standard error of the mean.

The mean and median levels of UF_SG_ (mg/L) by sex, age, and CWF status are presented in Table 1. As expected, participants living in a fluoridated region had significantly higher UF_SG_ than those living in a non-fluoridated region for each of the six age groups (Figure 2).

**Figure 3:**
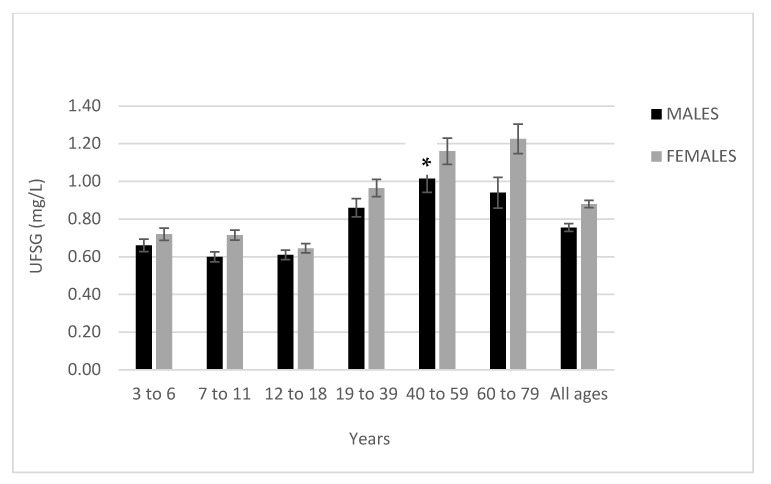
Mean UF_SG_ levels by age group and sex. Error bars represent the standard error of the mean. * Depicts significant difference between males and females (*p* < 0.05).

When collapsed across age groups, UF_SG_ concentration was 82% higher among participants who received fluoridated drinking water (*M* = 1.06 mg/L, *SD* = 0.83) than among those who did not (*M* = 0.58 mg/L, *SD* = 0.47), *t* = −13.7, *p* < 0.01. Females had higher UF_SG_ levels than males across all age groups (Table 1; Figure 3), though the differences were only significant for females aged 60 to 79 (females: *M* = 1.16 mg/L, *SD* = 1.00; males: *M* = 0.94 mg/L, *SD* = 0.97), *t* = −2.0, *p* = 0.039. Collapsing across age and CWF status, females had significantly higher UF_SG_ concentration (*M* = 0.89 mg/L, *SD* = 0.76) than males (*M* = 0.77 mg/L, *SD* = 0.67), *t* = −3.4, *p* < 0.01.

Females aged 60 to 79 living in fluoridated regions had the highest level of UF_SG_ at 1.56 mg/L, followed by females aged 40 to 59 at 1.51 mg/L. Males aged 7 to 18 living in non-fluoridated regions had the lowest levels of UF_SG_ at 0.43 mg/L.

### 3.3. Differences in UF_SG_ by Drinking Water Habits

In fluoridated regions, UF_SG_ concentration was significantly higher among participants who report drinking primarily tap water (*M* = 1.09 mg/L, *SD* = 0.86) than among those who report drinking primarily bottled water (*M* = 0.95 mg/L, *SD* = 0.67), *t* = 2.06, *p* = 0.04. In non-fluoridated regions, UF_SG_ concentration was similar among participants who report drinking primarily tap water (*M* = 0.58 mg/L, *SD* = 0.48) compared with those who report drinking primarily bottled water (*M* = 0.56 mg/L, *SD* = 0.39), *t* = 0.72, *p* = 0.47. In fluoridated regions, nearly all participants reported receiving municipal tap water and very few reported using a private well (*t*-tests for this comparison were not permitted due to Statistics Canada sample size requirements for data release). In non-fluoridated regions, UF_SG_ concentration was significantly higher among participants who received their water from a private well (*M* = 0.73 mg/L, *SD* = 0.57) than among those who received municipal tap water (*M* = 0.54 mg/L, *SD* = 0.43), *t* = −3.53, *p* < 0.01.

### 3.4. Differences in UF_SG_ by Dental Product Use

The UF_SG_ concentration among participants who report using fluoridated products at home (*M* = 0.88 mg/L, *SD* = 0.77) was similar among those who do not use fluoridated products at home (*M* = 0.86 mg/L, *SD* = 0.89), *t* = 0.23, *p* = 0.82. However, participants who reported that they used a fluoride-containing dental product (such as toothpaste) less than six hours before the urine sample was collected had significantly higher levels of UF_SG_ (*M* = 0.94 mg/L, *SD* = 0.79) than those who reported using a fluoride-containing dental product six or more hours before the sample collection (*M* = 0.74 mg/L, *SD* = 0.61), *t* = 4.0, *p* < 0.001. Table 2 compares UF_SG_ across age groups based on the recency of their use of fluoridated products.

Children aged 3 to 6 years old who used fluoride-containing products within six hours of the sample collection (*M* = 0.83, *SD* = 0.62) had significantly higher levels of UF_SG_ than children who used a fluoridated dental product more than six hours before sample collection (*M* = 0.61, *SD* = 0.39), *t* = 3.60, *p*< 0.001. Similarly, adults aged 19 to 39 years old (*M* = 1.01, *SD* = 0.70, *t* = 2.6, *p* = 0.01) and aged 40 to 59 years old (*M* = 1.27, *SD* = 0.86, *t* = 2.3, *p* = 0.02) who used fluoride-containing dental products within six hours of the sample collection had significantly higher levels of UF_SG_ than those who did not.

### 3.5. Differences in UF_SG_ by Tea Drinking Habits

Participants who reported that they drank green, black, or white tea within 24 h of the urine sample collection had 51% higher levels of UF_SG_ (*M* = 1.31 mg/L, *SD* = 1.04) than those who did not (*M* = 0.87 mg/L, *SD* = 0.67), *t* = 5.6, *p* < 0.01. Furthermore, participants who reported that they typically drink two or more cups of green, black, or white tea at a time had significantly higher levels of UF_SG_ (*M* = 1.40 mg/L, *SD* = 1.15) than those who reported drinking one cup of tea (*M* = 0.93 mg/L, *SD* = 0.73), *t* = −4.1, *p* < 0.01.

### 3.6. Predictors of Urinary Fluoride Concentration

The results of the multiple linear regression model are presented in Table 3. Overall, the complete set of predictors in this model explained 27% of the variance in UF_SG_. The regression slope coefficient (*B*) represents the degree of change in the outcome variable (UF_SG_) for every one unit of change in the predictor variable. Tap water fluoride level, CWF, age, sex, BMI, smoking allowed in the home, tea consumption and recency of dental-product use were significant unique predictors of UF_SG_. There was a non-linear effect of age on UF_SG_ such that UF_SG_ remained relatively stable across childhood (ages 3 to 18) but increased over adulthood (ages 19 to 79). Tap water fluoride predicted UF_SG_ concentration such that for every 1 mg/L increase in tap water fluoride, there was an increase of 0.48 mg/L UF_SG_ (95% CI = 0.25 to 0.71), holding the other predictors and covariates constant. Compared with those who live in a non-fluoridated region, individuals who receive CWF have a 0.39 mg/L higher level of UF_SG_ (95% CI = 0.24 to 0.53), and compared to males, females have a 0.12 mg/L higher level of UF_SG_ (95% CI = 0.03 to 0.20), holding the other predictors and covariates constant. Further, BMI significantly predicted UF_SG_ concentration such that for every kg/m^2^ increase in BMI, there is a predicted decrease of 0.02 mg/L UF_SG_ (95% CI = −0.02 to −0.01).

Compared to those who allow smoking in their home, those who do not allow smoking in the home have a 0.25 mg/L lower level of UF_SG_ (95% CI = −0.41 to −0.09). Compared to individuals who do not drink tea, people who report drinking green, black, or white tea had a 0.13 mg/L higher level of UF_SG_ (95% CI = 0.03 to 0.22). Finally, individuals who did not use a fluoride-containing dental product near the time of urine sampling had a lower UF_SG_ level compared to those who did (*p* = 0.049). Household income, highest household education, ethnicity, primary source of drinking water (tap or bottle), and last fluoride treatment at dentist were not significantly and uniquely associated with UF_SG_. Age by sex and CWF by primary source of drinking water (tap or bottled) interactions were not significant; thus, these terms were not included in the model described above.

## 4. Discussion

In Canada and the United States, the recommended fluoride level in drinking water is 0.7 mg/L for community water fluoridation (CWF), although naturally occurring fluoride levels can exceed this standard in some regions. Given that drinking water is a main source of fluoride exposure for most individuals [6,7], this study sought to characterize differences in urinary fluoride adjusted for specific gravity (UF_SG_) as a function of CWF status, age, and sex. In this Canadian sample of 1629 individuals aged 3 to 79 years, we found that UF_SG_ concentration was 82% higher among participants receiving fluoridated drinking water (*M* = 1.06 mg/L, *SD* = 0.83) than those receiving non-fluoridated water (*M* = 0.58 mg/L, *SD* = 0.47). This difference is consistent with other Canadian studies reporting that individuals living in fluoridated regions have between 1.5 and 2 times greater UF concentration than individuals living in non-fluoridated regions [13,17,21,38]. Likewise, U.S. children and adolescents drinking fluoridated tap water had 36% higher plasma fluoride levels than those not consuming fluoridated water [20].

Our findings underscore the importance of reporting fluoride exposure levels or health outcomes associated with fluoride intake according to CWF status, especially in countries where some individuals receive fluoridated tap water and others do not. For example, the Canadian Health Measures Survey 2007–2009 Oral Health Component reports a national prevalence of less than 13% for mild to more severe forms of dental fluorosis, a permanent discoloring of the tooth enamel associated with excess fluoride intake during enamel formation [39]. By not reporting prevalence of dental fluorosis as a function of CWF, this would obscure the population at greatest risk of showing enamel fluorosis, especially when only about one-third of Canadian households receive fluoridated tap water [1].

Tap water fluoride predicted UF_SG_ concentration such that every 1 mg/L increase in tap water fluoride is associated with an increase of 0.48 mg/L UF_SG_ after covariate adjustment. The association between tap water fluoride and UF_SG_ was largest for adults and smallest for children, consistent with studies showing that water and other beverages account for approximately 60–78% of dietary fluoride intake among adults, but only 40% of dietary fluoride intake for children 1 to 10 years old [7,40]. Other important sources of fluoride intake in children may include fluoride-containing dental products or foods that are high in fluoride, such as grapes/raisins, shellfish/fish, strained chicken with broth, and processed chicken. White grape juice has high fluoride levels (mean of 1.45 mg/L) due to the use of cryolite as a pesticide on grapes whereas processed chicken can have high fluoride due to mechanical deboning which leaves some skin and residual bone particles in the meat [7]. However, researchers who conducted the National Health and Nutrition Examination Survey (NHANES) with U.S. children and adolescents did not find an association between plasma fluoride levels and fluoride-rich foods and beverages, with the exception of tea consumption [20]. It is possible that fluoride from dietary sources is less bioavailable than fluoride found in tea [41].

Regarding age-related differences in urinary fluoride excretion, children and adolescents had lower levels of urinary fluoride compared with adults. Previous studies have found that young children (aged 1 to 4 years) have a higher daily intake of fluoride relative to their body weight from various sources than adults, regardless of fluoridation status [5]. Lower urinary fluoride excretion among children reflects increased fluoride absorption due to skeletal growth [42,43] and reduced elimination of fluoride through the kidney relative to adults [44]. In contrast, women aged 40 years and above and living in fluoridated regions had the highest urinary fluoride level. These findings are consistent with results of the Canadian Health Measures Survey (CHMS) Cycle 3 Biomonitoring Report [12]. Older women have higher UF_SG_ than men due to increased fluoride release from bone after menopause [45,46], as well as greater tea consumption [47]. These findings are of public health significance given that chronic exposure to fluoride can change the properties of bone and contribute to skeletal fractures [40], especially post-menopause, with increasing bone loss due to reduced steroid production [48]. A prospective cohort study from Sweden reported a 50% increased risk of hip fractures among postmenopausal women who had higher levels of urinary fluoride [40].

Participants who reported drinking green, black, or white tea had a 0.13 mg/L higher level of UF than those who did not drink tea, controlling for covariates_._ This finding is consistent with previous research demonstrating the large contribution of tea intake to urinary fluoride [17,47,49,50,51] and bone fluoride levels [46]. Tea plants are capable of hyper-accumulating fluoride from the soil into their leaves, particularly if the tea is grown in acidic soil [47]. Pregnant women who are daily tea drinkers have significantly higher levels of urinary fluoride levels compared to those who rarely consume green, black, white, or oolong tea [51]. Till et al. [17] found that black tea, but not green tea, accounted for approximately 5% of the variance in urinary fluoride levels measured in pregnant women. Higher levels of fluoride have been found in black teas compared to white or green teas, and when teas are steeped for longer periods of time (30 min of brewing versus 5 min; [47]).

We also found that participants who reported using a fluoride-containing dental product (such as toothpaste or mouthwash) less than six hours before the urine sample was collected had higher levels of UF_SG_ than those who did not. As expected, this finding was especially strong for children ages 3 to 6. This finding is consistent with previous research on the impact of accidental toothpaste ingestion on urinary fluoride levels in young children whose spitting reflex is not fully developed [52,53]. Our findings also concur with previous studies showing increases in urinary fluoride in children aged 5 to 8 years after using fluoride-containing dental varnishes [54]. Taken together, urinary fluoride level varies substantially depending on participant behaviour prior to sampling and may not be representative of long-term fluoride exposure.

Fluoride metabolism can be modified by exposure to tobacco smoke due to enzyme induction by compounds found in cigarettes. Individuals who smoke cigarettes have markedly higher plasma fluoride concentrations compared with non-smokers following use of a fluoridated anesthetic [36]. Previous research has also revealed that individuals who smoke cigarettes have higher rates of dental and skeletal fluorosis and higher levels of urinary fluoride [49,55]. Likewise, we found that individuals who are exposed to tobacco smoke in the home have 0.25 mg/L higher UF_SG_ compared with those who live in homes in which smoking is not permitted inside the home. However, given the small sample of individuals in our study who allowed smoking inside their home (9%), further research is needed to understand how exposure to second-hand-smoke may affect fluoride metabolism.

Susceptibility to fluoride depends on the level and chronicity of exposure from ingesting fluoridated water, tea, or other sources. Currently, approximately 3% of Europeans, 39% of Canadians, and 73% of Americans on public water supplies receive community water fluoridation [1,2]. Consumption of optimally fluoridated water (i.e., 0.7 mg fluoride per liter of water) accounts for approximately 40% to 70% percent of daily fluoride ingestion [7] making it the single largest source of chronic fluoride exposure for those receiving CWF. Using the tap water consumption values documented in the 2019 Environmental Protection Agency report [56], fluoride exposure from ingesting fluoridated water ranges from 0.011 to 0.013 mg/kg/day for adults. Notably, actual fluoride intake would be higher if intake from ingesting commercial beverages made with community water or other sources (e.g., tea) was factored in. It has been estimated that approximately two thirds of the world’s population drinks tea [57], which is thus another notable source of fluoride for many individuals worldwide. Susceptibility to fluoride is also crucially dependent on timing of exposure (i.e., life stage), and other biological factors, such as genetics, renal impairment, and iodine deficiency. Fluoride intake can be as high as 0.13 to 0.2 mg/kg/day for infants who are fed formula made with fluoridated water and are at the 95th percentile for water consumption; these values exceed the upper limit for fluoride intake for infants younger than four months (0.1 mg/kg/day) [58]. The fetal period is also considered a critical period of susceptibility to fluoride’s neurotoxic effects [38,59]. Regulatory agencies should consider fluoride intake from all sources in pregnant women, and especially women living in fluoridated communities where fluoride exposure levels would be higher.

Strengths of this study include assessment of various dietary and dental sources of fluoride, and individualized measures of fluoride in tap water and urine samples collected in a large sample of children and adults living in regions with and without CWF. However, use of one spot urine sample may have introduced error given the short half-life of fluoride and the impact of consuming tea or inadvertent ingestion of fluoridated dental products prior to urine sampling. Further, we did not measure the amount of water each participant consumed per day to estimate total exposure (intake) from tap water consumption. Another limitation is that our sample was predominantly white (73%), which precluded investigating differences in fluoride excretion by ethnicity as reported by other studies [60]. Finally, we did not have information about whether participants moved from a non-fluoridated area to a fluoridated area in their lifetime, which could affect fluoride levels that are stored in bone and released at a later age.

In conclusion, urinary fluoride levels are substantially higher among individuals across the lifespan living in areas with fluoridated water. Other common sources of fluoride included tea intake and recency of dental product use. We also observed sex differences in urinary fluoride levels among older women, perhaps reflecting biologic differences (e.g., role of estrogen in bone remodeling after menopause) or gender-based differences (e.g., higher tea intake in women compared with men). Given growing concerns about adverse health effects of fluoride exposure to the developing fetus [38,59], young children [22,61], and in other vulnerable populations, including those who are iodine deficient [62] or post-menopausal [40], further research is needed to investigate how specific sources of fluoride exposure and timing of exposures may relate to health outcomes across the lifespan.

## Figures and Tables

**Table 1:** UF_SG_ (mg/L) by sex, age group, and CWF status, with *t*-tests comparing UF_SG_ for individuals living in fluoridated and non-fluoridated regions by age group.

Age Group and Sex	Fluoridated (N = 860)					Non-Fluoridated (N = 780)					*p*-Value
	*n*	Mean	5th, 95th Percentile	SD	Median	*n*	Mean	5th, 95th Percentile	SD	Median	*p*
Age 3 to 6	180	0.82	0.27, 1.68	0.59	0.70	185	0.57	0.18, 1.29	0.41	0.46	<0.001
Male	93	0.80	0.27, 1.58	0.57	0.70	86	0.52	0.16, 1.16	0.32	0.46	
Females	87	0.83	0.27, 1.71	0.61	0.72	99	0.61	0.21, 1.43	0.47	0.46	
Age 7 to 11	145	0.88	0.40, 1.52	0.78	0.72	123	0.46	0.18, 1.10	0.28	0.38	<0.001
Male	77	0.77	0.40, 1.27	0.32	0.72	57	0.43	0.18, 1.20	0.27	0.38	
Female	68	1.00	0.40, 2.09	1.07	0.75	66	0.43	0.18, 1.01	0.25	0.38	
Age 12 to 18	165	0.79	0.34, 1.52	0.37	0.70	140	0.47	0.21, 0.90	0.34	0.40	<0.001
Male	79	0.79	0.34, 1.50	0.35	0.72	67	0.43	0.18, 0.87	0.27	0.38	
Female	86	0.78	0.36, 1.60	0.38	0.67	73	0.51	0.23, 0.93	0.44	0.57	
Age 19 to 39	139	1.24	0.44, 2.47	0.78	1.05	119	0.59	0.23, 1.22	0.38	0.51	<0.001
Male	64	1.22	0.42, 2.47	0.78	1.05	58	0.50	0.23, 1.08	0.28	0.44	
Female	75	1.25	0.49, 2.66	0.77	1.05	61	0.68	0.27, 1.22	0.44	0.57	
Age 40 to 59	104	1.46	0.48, 3.61	0.86	1.27	102	0.73	0.21, 2.09	0.66	0.50	<0.001
Male	42	1.38	0.55, 3.42	0.84	1.16	55	0.65	0.19, 1.71	0.62	0.49	
Female	62	1.51	0.42, 3.61	0.88	1.34	47	0.81	0.21, 2.28	0.70	0.59	
Age 60 to 79	121	1.47	0.42, 3.80	1.24	1.06	106	0.72	0.23, 2.09	0.64	0.54	<0.001
Male	58	1.37	0.36, 4.37	1.39	1.00	49	0.51	0.21, 1.03	0.33	0.44	
Female	63	1.56	0.46, 3.80	1.09	1.14	57	0.89	0.27, 2.66	0.78	0.65	
Total	854	1.06	0.36,2.47	0.83	0.84	775	0.58	0.19, 1.43	0.47	0.46	<0.001
Male	413	1.00	0.34,2.09	0.78	0.80	372	0.51	0.18, 1.08	0.37	0.43	
Female	441	1.12	0.38,2.66	0.86	0.86	403	0.64	0.21, 1.60	0.53	0.49	

**Table 2:** *T*-tests comparing UF_SG_ for individuals who have and have not used a fluoridated product recently, by age group.

Age Group	Used a Fluoridated Product Less Than 6 h before Sample Collection				Used a Fluoridated Product 6 or More Hours before Sample Collection				*p*
	*n*	Mean (95% CI)	SD	Median	*n*	Mean (95% CI)	SD	Median	
Age 3 to 6	94	0.81 (0.72, 0.93)	0.62	0.72	144	0.62 (0.56, 0.67)	0.39	0.51	<0.001
Age 7 to 11	88	0.64 (0.58, 0.69)	0.32	0.57	129	0.68 (0.55, 0.69)	0.67	0.53	0.78
Age 12 to 18	136	0.65 (0.55, 0.64)	0.34	0.58	110	0.64 (0.54, 0.65)	0.37	0.55	0.97
Age 19 to 39	139	1.01 (0.83, 0.99)	0.70	0.84	86	0.83 (0.66, 0.84)	0.58	0.67	0.01
Age 40 to 59	93	1.27 (0.96, 1.18)	0.86	1.10	61	0.93 (0.73, 1.00)	0.85	0.67	0.02
Age 60 to 79	103	1.29 (1.02, 1.40)	1.29	0.91	53	1.07 (0.81, 1.14)	0.85	0.84	0.06
Total	653	0.94	0.79	0.72	583	0.74	0.61	0.59	<0.001

**Table 3:** Linear regression predicting UF_SG_ (mg/L).

Predictor	B	95% CI	*p*
Water fluoride (mg/L)	0.48	0.25, 0.71	<0.01
CWF status (ref: non-fluoridated area)	0.39	0.24, 0.53	<0.01
Age	0.03	0.01, 0.04	<0.01
Age^2^	−0.02	−0.03, −0.01	<0.01
Sex (ref: male)	0.12	0.03, 0.20	0.01
BMI	−0.02	−0.02, −0.01	<0.01
Tea consumption (ref: none)	0.13	0.03, 0.22	0.01
Smoking allowed in the home (ref: yes)	−0.25	−0.41, −0.09	<0.01
Ethnicity (ref: white)	−0.07	−0.17, 0.04	0.20
Household education (ref: less than bachelor’s degree)	0.05	−0.05, 0.14	0.35
Income (per $100,000 CND)	−0.04	−0.10, 0.02	0.17
Primary source of drinking water (ref: tap)	−0.01	−0.18, 0.16	0.87
Last fluoride treatment at dentist (ref: < 3 months ago)	−0.03	−0.15, 0.08	0.58
Time since use of a fluoride-containing dental product (ref: < 6 h ago)	−0.09	−0.18, 0.00	0.05

Note. *N* = 900, *R*^2^ = 0.27, *F*(15, 883) = 22.16, *p* < 0.01.

## Data Availability

Restrictions apply to the availability of these data. Data was obtained from Statistics Canada and require an application to Statistics Canada for access. More information on how to apply for access can be found at: https://www.statcan.gc.ca/eng/microdata/data-centres/forms, accessed on 7 June 2021.

## Supplementary Materials

The following are available online at https://www.mdpi.com/article/10.3390/ijerph18126203/s1, Table S1: Fluoridation status by site in Cycles 3 of the CHMS.
