# Supplementary material for: Urinary Fluoride Levels among Canadians with and without Community Water Fluoridation
Source: Int J Environ Res Public Health. 2021 Jun 8;18(12):6203. doi: 10.3390/ijerph18126203 (PMC8226595; doi:10.3390/ijerph18126203)
Supplement: Supplementary file 1 [file ijerph-18-06203-s001.zip › ijerph-1243385-supplementary.pdf]

Supplemental Table S1.

Fluoridation status by site in Cycles 3 of the CHMS

Fluoride added to public water	Site	Region	Rationale for Classification
**No**	East Montreal	Quebec	Online sources including a map of water treatment plants in Montreal confirms that the water treatment plant that services East Montreal (Atwater et Des Baillets) does not add fluoride.
	South Central Laurentians	Quebec	This site is comprised of a number of regional municipalities likely including Les Laurentides, Argenteuil, Deux-Montagnes, La Rivière-du-Nord, Les Pays-d'en-Haut, Mirabel, Thérèse-De Blainville. Municipal government employees from Deux-Montagnes, Les Pays-d'en-Haut and Mirabel. Municipalities in Thérèse-De Blainville stopped fluoridation in 2002 according to http://blogues.radio-canada.ca/rive-sud/2013/07/03/buvez-vous-de-leau-fluoree/
	Orillia	Ontario	A presentation by the regional health authority confirms that Orillia has never added fluoride (http://www.simcoemuskokahealth.org/docs/default-source/topic-oralhealth/cwffactsheetpublic)
	Victoria-Saanich	British Columbia	The Greater Victoria Drinking Water Quality Annual Reports confirm that fluoridate is not added to the drinking water in Greater Victoria.
	Vancouver	British Columbia	The Metro Vancouver website (drinking water treatment processes page) confirms that fluoride is not added to the water supply (http://www.metrovancouver.org/services/water/quality-facilities/facilities-processes/treatment-process/Pages/default.aspx)
**Yes**	Oshawa-Whitby	Ontario	Annual water treatment reports confirm the addition of fluoride to the municipal drinking water: https://www.durham.ca/en/living-here/resources/Documents/WaterandSewer/OWAWaterQualityReport2016.pdf
	North Toronto	Ontario	All areas of Toronto add fluoride to their drinking water, as stated by: https://www.toronto.ca/311/knowledgebase/kb/docs/articles/toronto-water/water-treatment-and-supply/operations-efficiency/fluoridation-of-city-drinking-water-fluoride-levels.html
	Brampton	Ontario	The city of Brampton is part of the region of Peel, which adds fluoride across the region. See: http://www.peelregion.ca/health/fluoride/
	Lethbridge	Alberta	Lethbridge has added fluoride to the drinking water since 1974. More information is available from: https://www.lethbridge.ca/living-here/water-wastewater/Documents/Consumer%20Confidence%20Report%202016.pdf
	Halifax	Nova Scotia	All water treatment plants in Halifax add fluoride to their drinking water supply, as confirmed by: https://www.halifax.ca/home-property/halifax-water/water-services
**EXCLUDED**			
	Southwest Montérégie	Quebec	The municipalities contained within this site were unclear and likely contained a mix of both fluoridated and non-fluoridated municipalities. Since postal code data were not available, sites could not be divided into separate areas and the whole site was therefore excluded.
	West Montreal	Quebec	Part of the region is supplied by a water treatment plant that adds fluoride, and another part of the region is supplied by a water treatment plant that does not add fluoride. Since postal code data were not available, sites could not be divided into separate areas and the whole site was therefore excluded.
	Windsor	Ontario	Windsor stopped fluoridation on January 29 2013, which was during the data collection for Cycle 3. About 78% of tap water samples from this site were collected after fluoridation was discontinued.

(stopped Jan 29, 2013)		
Southwest Calgary (stopped in 2011)	Alberta	Calgary began adding fluoride to the drinking water supply in 1991, and stopped fluoridation in May 2011. For more information see: http://www.calgary.ca/UEP/Water/Pages/Drinking-water/Fluoride.aspx. Since fluoridation stopped just before Cycle 3 data collection for this site, the tap water fluoride average for this site was almost 3 times higher than for other non-fluoridated sites. Therefore, this site was excluded from analyses using city fluoridation status as a variable.
Brantford-Brant County	Ontario	The City of Brantford Water System adds fluoride to their municipal water, as described in their 2013 Annual Report available from: http://www.brantford.ca/residents/health/water_quality/waterquality/Pages/MOEAnnualReport.aspx Brant County, however, is a rural area in which many individuals are on well water. According to their water treatment report from 2013, the level of fluoride in the region ranges from 0.05 mg/L to 0.25mg/L: https://www.brant.ca/en/invest-in-brant/resources/Area_Studies_Documentation/Northwest-Paris-Area-Study/Azimuth-Final-Brant-Water-Supply-Study-Report-April-2014.pdf The average tap water level for each site in Cycle 3 was calculated, and the average level of fluoride in tap water was 3 times lower for this site than for the average of all fluoridated sites. Any fluoridated site with an average tap water level less than 0.3 mg/L was excluded from classification as a fluoridated site.
Kent County	New Brunswick	The Kent County Water Authority States that they do add fluoride to the water at the range of 0.66 - 1.0 ppm: https://kentcountywater.org/config/rpts-qual/CCR2012.pdf. The county consists of two towns and three small villages, some of which may receive well water. The average tap water level for each site in Cycle 3 was calculated, and the average level of fluoride in tap water was 3 times lower for this site than for the average of all fluoridated sites. Any fluoridated site with an average tap water level less than 0.3 mg/L was excluded from classification as a fluoridated site.
